# Literary Notes

**Published:** 1910-06

**Authors:** 


### LITERARY NOTES

THE *New England Medical Monthly* published at Danbury, Conn., has been purchased by the Annals Publishing Company of Boston, and will be combined with the *Annals of Medical Practice*. Dr. Francis D. Donoghue formerly editor of the *Annals*, will continue in charge of the consolidated journals.

THE *American Journal of Physiologic Therapeutics* has made its appearance, the first number bearing date May 1910. It is to be published bimonthly by Dr. Henry R. Harrower, 72 Madison Street, Chicago. It presents a fine appearance in dress, and in material it contains much of value.

"A Few Genitourinary Facts" is the title of a little brochure recently issued by Dr. J. Henry Dowd, of Buffalo. It is a valuable aid to the diagnosis of many faults or lesions of the genitourinary tract, and a reminder of many conditions liable to be overlooked in the examination. The booklet can be obtained of the author, 40 North Pearl Street, Buffalo, N. Y.

